# A multicenter study on rates and risk factors of proliferative vitreoretinopathy in a low-income economy

**DOI:** 10.1371/journal.pone.0316058

**Published:** 2025-07-24

**Authors:** Ogugua Ndubuisi Okonkwo, Martha-Mary Udoh, Olukorede Adenuga, Wilson Ovienria, Affiong Ibanga, Dennis Nkanga, Idris Oyekunle, Toyin Akanbi, Chineze Agweye

**Affiliations:** 1 Department of Ophthalmology, Eye Foundation Hospital & Eye Foundation Retina Institute, Lagos, Nigeria; 2 Department of Ophthalmology, University of Uyo Teaching Hospital, Akwa Ibom, Nigeria; 3 Department of Ophthalmology, University of Jos Teaching Hospital, Plateau, Nigeria; 4 Department of Ophthalmology, Irrua Specialist Hospital, Irrua, Edo, Nigeria; 5 Department of Ophthalmology, University of Calabar Teaching Hospital, Cross River, Nigeria; Kamuzu University of Health Sciences, MALAWI

## Abstract

**Objective:**

To assess the rate and identify risk factors for proliferative vitreoretinopathy (PVR) in Nigerian patients with rhegmatogenous retinal detachment (RRD).

**Methods and analysis:**

Multicenter, prospective, cross-sectional study of consecutive RRD patients attending four ophthalmic clinics, recruited over a period of twelve months. Biodata, presenting complaints, symptom duration, best corrected visual acuity, extent of RRD, status of macular attachment, presence, and grade of PVR and fellow eye were assessed. Data was analyzed using SPSS version 22 (Pearson Chi-Square test, Mann-Whitney U test, Fisher’s Exact test, and logistic regression analysis).

**Results:**

154 of 174 eyes with RRD were included in the final analysis, and 53/154 (34.4%) developed PVR. Twenty eyes (11.5%) had media opacity, and detailed retinal examination was not possible (diagnosed via B-scan ultrasound). 154 RRD was analyzed. Therefore, the rate of all PVR was53/154 (34.4%). Grades of PVR: Grade D: 27 eyes (50.9%), Grade C: 19 eyes (35.9%), Grade B: 5 eyes (9.4%), and Grade A: 2 eyes (3.8%). The rate of advanced PVR grades D + C is 46/154 (29.8%). Forty-six of 53 PVR eyes (86.6%) had vision 3/60 or less (p < 0.001). Risk factors associated with PVR were extremes of age, i.e., < 25 and >66 years (especially older age (>66 years)), previous intraocular surgery, longer symptom duration, and RD features including 4-quadrant involvement, total RD, macular involvement by RD, and presence of fellow eye RD. Gender, Myopia, and trauma were not risks for PVR.

**Conclusion:**

Previous reports of PVR rates in Nigerians may have been underestimated. A multi-pronged approach, including research into adjuvant pharmacotherapy, can reduce PVR rates and improve treatment outcomes.

## Introduction

Proliferative vitreoretinopathy (PVR) is a clinical condition that complicates rhegmatogenous retinal detachment (RRD). PVR can occur before surgery or arise after the surgical repair of RRD. It is the most common cause of RRD repair failure and, therefore, a vision- damaging complication. Pathologically, PVR is characterized by the proliferation, growth, and contraction of cellular membranes within the vitreous cavity and on both sides of the retinal surface; intraretinal fibrosis also occurs [1,2]. Following a retinal break, the underlying retinal pigment epithelium (RPE) cells migrate into the vitreous cavity. These vitreal RPE cells play a significant role in PVR formation by interacting with glial cells, hyalocytes, macrophages, and fibroblasts in the vitreous, through a complex series of inflammatory responses. This results in the release of cytokines and growth factors, forming extracellular matrix proteins that assemble into membranes with contractile properties, which is characteristic of PVR [3]. Risk factors for PVR include preoperative RD characteristics such as large or multiple retinal breaks, including giant retinal breaks, prior infectious retinitis, intraocular inflammation, prolonged retinal detachment, aphakia, concurrent choroidal detachment, a large extent of retinal detachment, vitreous hemorrhage, and preoperative grade A or B PVR [4]. Intraoperative factors include vitreous or preretinal hemorrhage, excessive cryotherapy, pigment release during endo drainage, and external drainage of subretinal fluid complicated by vitreous loss. Smoking has been associated with an increased risk of PVR [4,5]. Xiang et al. reported findings from a systematic review on risk factors for postoperative PVR, utilizing a large number of studies and patients [5]. They identified the following risks for PVR: increased age, the presence of preoperative PVR, a higher risk for smokers compared to nonsmokers, the presence of vitreous hemorrhage, and aphakia or pseudophakia. Additionally, the presence of a macula- off, a larger extent of RD, and a longer duration of RD symptoms were noted as further risk factors [5]. Furthermore, aside from anatomical effects, PVR is known to have a harmful impact on vision.

The visual potential and prognosis following significant PVR are poor, and most eyes, despite the best efforts at retinal attachment, cannot see beyond 6/60 (20/200 equivalent) [6,7]. Due to delays in the presentation and repair of RRD in low-income countries, the reported rates of PVR are high, surpassing those in developed countries where access to RRD repair is readily available. Reported PVR rates in developed countries range from 5% to 10% [2]. Retrospective studies from single centers in sub-Saharan Africa report PVR rates that vary widely from 16.5% to up to 60% [8,9]. Investigating PVR rates in low-income countries and the West African region will provide a reference point for estimating the impact and effectiveness of interventions aimed at reducing the regional burden of PVR in the future. Consequently, we conducted a multicenter, prospective, hospital-based study to evaluate PVR rates in RRD and assess the risk factors for pre-operative PVR at the initial clinic visit.

## Materials and methods

We performed a multicentre, prospective, cross-sectional study involving patients attending ophthalmic clinics in four hospitals in Nigeria. Nigeria is subdivided into six zones that are named broadly according to their location: South and North, and then the South is further subdivided into East, South, and West. The North is further subdivided into East, Central, and West. There was one private and three public hospitals that had busy ophthalmic clinics. The hospitals were one in the North Central, two in the South South, and one in the South West regions of Nigeria. The twelve-month study period was from 1st April 2019–30th March 2020. All ophthalmic patients who presented at each of the four study sites were screened to determine if they had an RRD and if this was complicated by the presence of PVR.

Eligibility Criteria: All patients who were diagnosed with RRD were eligible for inclusion in the research and there were no exclusion criteria. Patients of all demographics were included and there was no age restriction. In 1983, the Retina Society Terminology Committee unified the definition and recognized four stages of PVR: A, B, C, and D, in order of increasing severity, from minimal to more severe PVR [10]. Contractile elements in the pre- and subretinal spaces add a tractional component to the RRD.

While PVR grades A and B are considered less severe, grades C and D are classified as severe and advanced stages of the disease. Grade C PVR is characterized by full-thickness fixed retinal folds, whereas Grade D represents a funnel-shaped RRD with fixed retinal folds present in all four quadrants. In this study, PVR was grouped according to the four stages from A to D. The updated classification of PVR was not utilized in this study. This revised classification, which considered the prognostic effect of PVR grade, was proposed in 1991 by Machemer et al., [11] further categorizing PVR into anterior and posterior classifications based on the number of clock hours involved.

Therefore, eligible participants had PVR grades A, B, C, or D, and a detailed view of the fundus was possible. In patients whose fundal view was obstructed by an opacity, RRD could be diagnosed, but this eye was excluded from the calculation of the PVR rate. The diagnosis of RRD and grading of PVR were conducted by a retina specialist who served as the designated principal investigator for each of the four research sites. In patients where the fundal view was obstructed by an opacity, a B scan was utilized for diagnosis. Data was collected from consecutive RRD patients visiting retina or general ophthalmic clinics.

Initially, biodata was collected, and a presenting complaint or ophthalmic history of flashes of light, floaters, veil-like covering, or visual field defects was noted to suggest RRD. A comprehensive ocular examination was conducted, including visual acuity testing with a Snellen visual acuity chart. The best-corrected visual acuity (BCVA) for each eye was assessed and recorded. Legal blindness was defined as a BCVA of 3/60 (Snellen Acuity) or less [12]. Subsequently, slit lamp biomicroscopy was performed to evaluate the anterior segment of the eye and measure intraocular pressure. A dilated slit lamp fundoscopy was carried out using either a 90D non-contact lens or a 20D lens and binocular indirect ophthalmoscopy. If the fundal view was obscured due to media opacity, a B-mode ultrasound scan was conducted to evaluate the posterior segment.

Informed consent to participate in the study was obtained from all patients diagnosed with RRD and from those diagnosed with any of the four grades of PVR. The study was conducted according to the principles of the Helsinki Declaration, and ethical approval was received from the Eye Foundation Hospital – Health Research Ethics Committee (Reference Number: EFH-HREC/ 2019/02). Study participants provided written informed consent and were given the option to decline examination without any negative consequences for their eye care.

Visual acuity was categorized using the International Classification of Diseases (ICD-10). Retinal detachment characteristics and risk factors for PVR, including symptom duration, visual acuity with best correction (BCVA), the extent of retinal detachment, and macula status (attached or detached) were specifically assessed.

Information from each study subject was entered into a Microsoft Excel spreadsheet, and the data from each of the four study sites was electronically transmitted to the data collation center at the end of each month. All data was collated at the center by a trained data analyst. The principal investigator at the study site was responsible for ensuring the accuracy and integrity of the data, timely reporting, and monthly transmission.

The data was analyzed using SPSS version 22. Categorical variables were expressed as frequency and percentage. The duration of symptoms was described as median and interquartile range.

Crosstabulation with Pearson’s Chi-Square was done to determine the association between categorical variables. The Mann-Whitney U test was used to compare the duration of symptoms between PVR and non-PVR eyes. Fisher’s exact test was applied when low or zero counts were present in some categories. Logistic regression analysis was done using simple logistic regression. Test for statistical significance was calculated, and values < 0.05 were considered significant.

## Results

One hundred and seventy-four (174) eyes of 167 patients, 117 (70.1%) males and 50 (29.9%) females diagnosed with RRD were the sample for this study.

The demographics and vision of PVR eyes are represented in Table 1. Of the 174 eyes, 53 (30.5%) eyes had PVR, and 101 (58%) had no PVR. We could not determine the presence or absence of PVR in 20 eyes (11.5%) because the view of the fundus was inadequate for detailed retina examination and was obscured by media opacity. The diagnosis of RRD was made with the aid of ultrasonography in these 20 eyes. Therefore, in this study, 154 RRD eyes were used for the analysis performed for PVR. Of the 53 eyes diagnosed to have PVR, a majority had Grade D, 27 eyes (50.9%). Grade C was present in 19 eyes (35.9%), while grades B and A occurred in 5 eyes (9.4%) and 2 eyes (3.8%), respectively. Therefore, the overall rate of “significant or advanced PVR” defined as grades C and D is 46 of 154 eyes (29.8%), and the rate of “all PVR” is 53 of 154 eyes (34.4%).

**Table 1:** Demographic, vision and distribution of PVR eyes.

Variable	Frequency (No of eyes)	Percent (%)
Age group		
25 years and below	11	20.8
26-45 years	18	34.0
46-65 years	14	26.4
46-65 years	10	18.9
Gender		
Male	34	64.2
Female	19	35.8
PVR Grades		
A	2	3.9
B	5	9.4
C	19	35.8
D	27	50.9
Visual Acuity (Snellen BCVA)		
6/12 and better	1	1.9
<6/12 − 6/18	1	1.9
<6/18 − 6/60	4	7.5
<6/60–3/60	1	1.9
<3/60	44	83
NPL	2	3.8

BCVA, best corrected visual acuity; PVR, proliferative vitreoretinopathy; NPL, no perception of light.

Forty-six out of 53 PVR eyes (86.6%) had a vision of 3/60 or less at presentation, and there was a statistically significant association between PVR and vision of 3/60 or less (p < 0.001). On regression analysis, PVR was associated with a 42% probability of vision 3/60 or less **(β = −0.314, OR=0.730, 95% CI = 0.14–3.85, p = 0.711)**. PVR grade D was associated with a 95% probability of vision 3/60 or less **(β = 2.907, OR=18.310, 95% CI = 2.41–139.39, p = 0.005**).

Nineteen of the 53 PVR eyes had undergone previous intraocular surgery. Cataract surgery was the most common surgery in ten (52.6%) of the 19 eyes. Three eyes (15.8%) had undergone couching of the lens. Fifteen eyes (28.3%) had previous eye trauma. Seven (46.7%) of the 15 eyes had blunt eye injuries. There was an ocular history in twenty-one fellow eyes of PVR cases, and nearly half of the fellow eyes had vision 3/60 or less and had phthisis bulbi, Table 2.

**Table 2:** Details of ocular diagnosis in the fellow eye of PVR eyes.

Ocular diagnosis	Frequency (No of eyes)	Percent (%)
Aphakia/subluxated IOL	2	9.5
Corneal opacity	2	9.5
Blindness/ phthisis bulbi	10	47.6
Cataract	6	28.6
Perripheral retinal degeneration	1	4.8
Total	21	100

Associations and risk factor assessment for PVR:

We examined some presenting clinical features in RRD patients for significant associations and risk factors for PVR.

Generally, there was significant age variation between PVR and No PVR eyes, p = 0.035. Twenty-one percent of eyes in the PVR group were 25 years or less compared to 12% in the No PVR group, suggesting that the younger age had more PVR, Table 3. Furthermore, the elderly, > 66 years, accounted for 19% of the PVR eyes, and only 6% of the No PVR eyes, suggesting that older age also had more PVR. There were three times more elderly RRD patients in the PVR group than in the No PVR group (19% versus 6%). In summary, extremes of age, < 25 years, and especially > 66 years are at risk of PVR, while the middle-aged did not show an increased risk of PVR.

**Table 3:** Associations and risk factors of PVR.

	PVR eyes	No PVR eyes	Pearson Chi 2(P < 0.05)
	N, %	N, %	P Value
25 years and below	11 (20.8%)	12 (11.9%)	0.035
26-45 years	18 (34.0%)	33 (32.7%)	
46-65 years	14 (26.4%)	50 (49.5%)	
>66 years	10 (18.8%)	6 (5.9%)	
Total	53 (100%)	101 (100%)	
Previous intraocular surgery	19 (35.8%)	19 (18.8%)	0.038
No previous intraocular surgery	34 (64.2%)	82 (81.2%)	
Total	53 (100%)	101 (100%)	
Previous eye trauma	15 (28.3%)	29 (28.7%)	0.837
No eye trauma	38 (71.7%)	72 (71.3%)	
Total	53 (100%)	101 (100%)	
Symptom duration*	4 months (IQR 12 months)	1.5 months (IQR 4.5 months)	0.001*
Myopia	9 (17%)	35 (34.7%)	0.048
No myopia	44 (83%)	66 (65.3%)	
Total	53 (100%)	101 (100%)	

Mann-Whitney U (P < 0.05).

Gender was not a risk factor for PVR. There was a higher proportion of males in both PVR and No PVR eyes. Males were 64.2% (34 eyes) and 75.2% (76 eyes), while females were 35.8% (19 eyes) and 24.8% (25 eyes) in the PVR and No PVR groups, respectively (p = 0.207).

A history of prior intraocular surgery and a longer duration of symptoms before presentation were significant risk factors for PVR (see Table 4). However, previous ocular trauma or myopia did not elevate the risk of PVR. Regarding myopia, the average degree of myopia for PVR eyes was −4.33D (SD 3.33D), which was comparable to that of No PVR eyes at −4.52D (SD 3.33D). PVR eyes were presented significantly later than eyes without PVR, as shown by the Mann-Whitney U test (p = 0.001). The median duration of symptoms in PVR eyes was 4 months (IQR – 12 months), compared to 1.5 months (IQR – 4.5 months) in No-PVR eyes.

**Table 4:** Comparison of retinal detachment presenting features in eyes with PVR versus No PVR.

	PVR	No PVR	Pearson Chi^2^ (P < 0.05)
	N (%)	N(%)	P Value
Previous intraocular surgery	19 (35.8%)	19 (18.8%)	0.038
No previous intraocular surgery	34 (64.2%)	82 (81.2%)	
Total	53 (100%)	101,(100%)	
Previous eye trauma	15 (28.3%)	29 (28.7%)	0.837
No eye trauma	38 (71.7%)	72 (71.3%)	
Total	53 (100%)	101 (100%)	
Symptom duration	4 months (IQR 12 months) m	1.5 months (IQR 4.5 months)	0.001
Myopia	9 (17%)	35 (34.7%)	0.048
No myopia	44 (83%)	66 (65.3%)	
Total	53 (100%)	101 (100%)	

There were more eyes with visual acuity 3/60 or less in PVR grade C (15 eyes, 34.1%) and grade D (24 eyes, 54.4%). However, visual acuity did not differ significantly between the four PVR grades, Table 5.

**Table 5:** Details of visual acuity in PVR eyes.

	Grade A	Grade B	Grade C	Grade D	Fisher’s exact test (P < 0.05)
					P Value
6/12 and better	0	0	1(100%)	0	0.286
<6/12–6/18	0	0	1(100%)	0	
<6/18–6/60	0	1 (25%)	2(50%)	1(25%)	
<6/60–3/60	0	1(100%)	0	0	
<3/60	2(4.5%)	3(6.8%)	15 (34.1%)	24(54.5%)	
NPL	0	0	0	2(100%)	

Fisher’s exact (P < 0.05).

The four RRD characteristics examined significantly impacted PVR. The quadratic involvement of RD, the extent of RD, macular involvement by RD, and the occurrence of fellow eye RD were all significant RRD risk factors for PVR (see Table 6). PVR eyes showed a higher proportion of 4-quadrant involvement, with double the rates of 4-quadrant RD compared to those without PVR (58.5% versus 25.7%). Additionally, PVR eyes exhibited more 3 and 4 quadrant involvement (77.4%) in PVR eyes (P < 0.001). Macular involvement by RD and the presence of RD in the fellow eye were more common in PVR compared to eyes without PVR, with P values of 0.035 and 0.009, respectively (see Table 6).

**Table 6:** Comparison of rhegmatogenous retinal detachment characteristics in eyes with PVR versus No PVR.

Quadrants of RRD	No PVR	PVR	Pearson Chi^2^ (P < 0.05)
			P Value
1 Quadrant	6, 5.9%	0, 0%	<0.001
2 Quadrants	44 (43.6%)	12 (22.6%)	
3 Quadrants	25 (24.8%)	10 (18.9%)	
4 Quadrants	26 (25.7%)	31 (58.5%)	
Total	101 (100%)	53 (100%)	
Extent of RD			
Subtotal RD	75 (74.3%)	22 (41.5%)	<0.001
Total RD	26 (25.7%)	31 (58.5%)	
Total	101 (100%)	53 (100%)	
Macular involvement			
Macula-off	80 (79.2%)	50 (94.3%)	0.035
Macula-on	21 (20.8%)	3 (5.7%)	
Total	101 (100%)	53 (100%)	
Fellow eye RD			
Absence of fellow eye RD	98 (97%)	44 (83%)	0.009
Presence of fellow eye RD	3 (3%)	9 (17%)	
Total	101 (100%)	53 (100%)	

On regression analysis, macula-off RD showed an increased likelihood of developing PVR (β = −0.256, OR=1.3, 95% CI = 0.27–6.22, p = 0.749). Previous intraocular surgery raised the possibility of developing PVR (β = 0.391, OR=0.68, 95% CI = 0.26–1.76, p = 0.423). Eyes with RD in the fellow eye (i.e., bilateral RRD at presentation) had higher odds of developing PVR (β = 1.57, OR=4.81, 95% CI = 0.04–1.00, p = 0.05). A longer duration of symptoms also increased the odds of developing PVR (β = 0.01, OR=1.01, 95% CI = 0.99–1.03, p = 0.202).

## Discussion

This is the first report of a multicenter study conducted in the West African region that investigates the rate of PVR in primary RRD, finding that advanced PVR occurs in 29.8% and any PVR is present in 34.4% of RRD eyes. This research also explored the role of RD-specific and non-ocular risk factors in the development of PVR. The high rate of pre-operative PVR among Nigerians and Africans, supported by this study, can be explained by an interplay of older age (>66 years), previous intraocular surgery, longer symptom duration, and RD characteristics, including 4-quadrant involvement, total retinal detachment, and macular involvement by RD, as well as the presence of RD in the fellow eye, all of which contribute to the risk of PVR in Nigerians.

This finding can be extrapolated to other African and low-income economies since the fundamental issues hinge on late presentation, limited access to ophthalmic care, and RD repair, and therefore, delayed surgery. Gender, myopia, and trauma were not risk factors for PVR.

Trauma has been reported by Eliott et al in a larger study as a risk factor for PVR [13]. In their study, Eliott et al, noted that a large number of eyes that suffered open globe injury developed PVR retinal detachment, different from our study report of a higher occurrence of blunt trauma. The difference in the type of trauma between the studies may be responsible for the non-risk factor status of trauma in our study. The high proportion of PVR eyes that had vision 3/60 or less in our study reflects the well-known vision-damaging effect of PVR.

The more advanced PVR grades C and D in this study are higher than the 16.5% previously reported by Okonkwo et al., who examined PVR rates in a series of surgical eyes among Nigerians [8]. Yorston reported a 17.5% rate of PVR C or worse in RRD eyes undergoing surgery in East Africa [14]. The advanced PVR rates reported by Okonkwo and Yorston are close but may have been underestimated since their reporting was based on surgical eyes, which could have been influenced by several factors, including refusal of surgery due to poor prognostic outcomes and financial constraints, as some patients must pay surgical fees out of pocket. Therefore, the advanced PVR rates reported in our study are likely higher than those in Okonkwo and Yorston’s report, as our study is not susceptible to the biases mentioned above. Reports of much higher rates of PVR in Africans also exist from Ethiopia (69%) and South Africa (33.3%) [15,16]. Additionally, a study on giant retinal tear RD in Nigerians reported an advanced PVR rate of 83% [17]. Tseng et al. reported preoperative advanced PVR in Venezuela as 26.9%, [18] which is slightly less than the 29.8% observed in our current study. The situation in Venezuela is similar to ours, with limited access to RRD treatment, which is directly and indirectly responsible for the high rates of PVR.

Because of the earlier presentation of RRD, pre-operative PVR rates in developed countries are much lower, translating to better visual outcomes following RRD surgery [19]. To facilitate earlier diagnosis and treatment of RRD in developing countries, access to ophthalmic/ retina care and patient education is critical, as pointed out by Tseng. We suggest that a three-pronged approach could help.

### This includes

1. Prioritizing fundus examination of patients suspected of having RRD, who present with suggestive symptomatology. The telemedicine approach using digital hand-held cameras or smartphones will help implement this strategy [20,21].
2. Training for retina care providers and equipping regional and secondary centers to repair noncomplex RRDs that can be managed with a scleral buckle and referring more complex cases to tertiary care eye hospitals equipped with vitreoretinal surgery capability.
3. Patient information campaigns on the need for timely ophthalmic visits, especially targeted at the at-risk population for RRD.

Several studies have examined risk factors associated with post-operative PVR and reported vitreous hemorrhage, PVR grade B, cigarette smoking, uveitis, macula involvement, increased retinal tear size (e.g., giant retinal tear), choroidal detachment, use of cryotherapy, air injection, and vitrectomy as significant risk factors [5,17,22–24].

Our study examined risk factors for pre-operative PVR and is the first study of this kind in Nigerians. Tseng identified long retinal detachment duration, poor initial visual acuity, and extensive retinal detachment as significant associations with pre-operative PVR. Additionally, vitreous hemorrhage and cataracts were linked to PVR severity. Our findings of prolonged symptom duration and extensive RD align with Tseng’s results. The presence of choroidal detachment is another pre-operative risk factor reported by Nagasaki.

Xiang et al. recently published a meta-analysis on risk factors for the formation of PVR after retinal reattachment surgery [5]. Although the current study investigated only the risk factors of pre-operative PVR, both studies (Xiang’s and the current study) share specific similar risk factors, confirming that the same promoters of pre-operative PVR are responsible for the formation of post-operative PVR. The similarities in risks between the two studies include increasing age, aphakia or pseudophakia, macula off, larger extent of RD, and longer duration of RD symptoms.

Risk identification aids in profiling patients’ diseases and determining which conditions require more PVR-specific attention. Pre-operative PVR has been shown to be a significant risk factor for the occurrence of post-operative PVR, according to Xiang and other researchers. Therefore, a sensible approach, if pre-operative PVR is present, is to explore the benefits of disease-modifying therapy to actively suppress the proliferation and inflammatory processes that characterize PVR, with the hope of achieving improved outcomes. Several pharmacotherapies have been utilized to treat or modify the outcome of PVR, though with limited success in human trials. These include steroids, 5-Fluorouracil (5FU), low molecular weight heparin, Mitomycin, and Daunomycin [25,26]. An effective therapy should provide a prophylactic advantage in eyes at higher risk for PVR, particularly those with pre-operative PVR. Identifying high-risk eyes can enable targeted treatment before or after the development of the early stages of PVR [26].

Though surgery, primarily pars plana vitrectomy with membrane peeling, is currently the cornerstone of PVR management, pharmacotherapy research for the prevention or prophylaxis of PVR has continued. The situation in low-income economies is further complicated by limited access to vitreoretinal surgery, due to the high cost of vitrectomy infrastructure and hardware, and the limited availability of necessary consumables. This calls for practical steps to reduce the occurrence of PVR since PVR converts the RRD from a simple to a more complex situation to manage.

Recently, promising reports in pharmacotherapy research, of the beneficial regression of PVR and improved outcomes following intravitreal use of Methotrexate, have emerged [27–29]. Also, Ranibizumab, has been shown to prevent PVR in rabbit models [30]. However, more evidence is required to establish the efficacy and safety of intravitreal methotrexate’s effect as prophylaxis or treatment [31–33]. An ideal pharmacotherapy should demonstrate reasonable safety, be uniformly effective in regressing PVR, prevent retinal re-detachment, and improve vision.

Weaknesses of this study include that, in some cases, self-reporting by the patient was relied upon, subjecting the information received to patient-related bias and inaccuracy. The PVR grading used was the older classification, which lacks prognostic value. Additionally, the effect of retinal break size on PVR was not assessed. While some study sites or centers could diagnose PVR, they lacked the necessary vitreoretinal capabilities for surgical treatment; therefore, reporting on the outcomes of surgical intervention is not possible at this time. Furthermore, some patients chose not to undergo surgery, resulting in not all patients receiving surgical interventions, and data on surgical outcomes were not retrieved. Moreover, the study’s design did not include research on outcomes. Future research on PVR in Nigeria aims to investigate the results of various interventions, including potential pharmacotherapy options. Lastly, a previous single-center report on PVR surgical outcomes reported anatomical benefits but limited visual benefits from PVR surgery in Nigerians [34].

Despite these weaknesses, the study has significant merits as a prospective, multicenter study with a reasonable sample of RRD patients. It provides valuable information for planning and advocacy in treating RRD complicated by PVR. Given that PVR is well established as a vision-damaging clinical condition, efforts must be made to reduce its rate in developing economies. Benefits can be gained from using teleophthalmology to improve the diagnosis time for RD, advocating to government and health agencies to invest in enhancing RD diagnosis and treatment services, and promoting patient education and campaigns aimed at higher-risk RD patients.

## Conclusion

High rates of advanced pre-operative PVR, 29.8%, and all PVR grades, 34.4%, occur in Nigerians. The PVR rate in this study is much higher than previous reports, which is likely underestimated, and is fueled by risk factors that include long duration before RRD presentation and repair, resulting in more macular off RD, and a higher extent of RD. Previous intraocular surgery and extremes of age are other risk factors for PVR. Our study findings are like those of pre- and post-operative risks from other regions. A multi-pronged approach will benefit efforts aimed at reducing PVR rates. This includes improving access to RD repair and increasing patient awareness through education. Next, the research and development of effective PVR pharmacotherapy will be beneficial in the clinical setting of pre-operative PVR or eyes at higher risk for post-operative PVR. Adjuvant use of such pharmacotherapy can serve as PVR prophylaxis, reducing the rates of post- operative progression or recurrence of PVR.

Key points from this study include the following;

What is already known on this topic: Proliferative vitreoretinopathy (PVR) is a vision-damaging consequence of rhegmatogenous retinal detachment (RRD), resulting in surgical failure and poor outcomes.

The rate of PVR in Africa and other low-income countries is unacceptably high, and efforts are needed to address this.

Previous studies on advanced PVR rates in Nigerians have been reported to be 16.5% of RRD eyes in a single center report of surgery patients.

What this study adds: This study suggests that previous estimates of the PVR rate in RRD are underestimated and the rate of advanced PVR is higher, 29.8%, than previous reports.

Our recent research is multicenter, prospective, and likely to be more accurate. It also highlights risk factors that can be used to profile patient population at risk of PVR including extremes of age and especially older age (>66 years), previous intraocular surgery, longer symptom duration, and RD features including 4 quadrant involvement, total RD, macular involvement by RD, and occurrence of fellow eye RD.

How this study might affect research, practice, or policy:

The findings of this study will be used as a benchmark to determine the effectiveness of interventions adopted to reduce the rate of PVR.

It will be used as advocacy to the public and relevant government agencies and institutions to improve detection and treatment of RRD and other retina diseases. It will also provide the at-risk attributes of RRD patients who should be targets for treatment or prophylaxis of PVR in the study population.
